# Exploring the mechanism of berberine treatment for atherosclerosis combined with non-alcoholic fatty liver disease based on bioinformatic and experimental study

**DOI:** 10.1371/journal.pone.0314961

**Published:** 2024-12-19

**Authors:** Shushu Wang, Kachun Lu, Liwen Lin, Gaijie Li, Yuxin Han, Zhichao Lin, Qingmin Chu, Kunsheng Wu, Peijian Liu, Guiting Zhou, Rui Peng, Chuanjin Luo

**Affiliations:** 1 The First Clinical Medical College, Guangzhou University of Chinese Medicine, Guangzhou, China; 2 Cardiology Center, The First Affiliated Hospital of Guangzhou University of Chinese Medicine, Guangzhou, China; 3 Shunde Hospital of Guangzhou University of Chinese Medicine, Foshan, China; 4 Guangdong Clinical Research Academy of Chinese Medicine, Guangzhou, China; University of Sri Jayewardenepura, SRI LANKA

## Abstract

Atherosclerosis (AS) and Non-alcoholic fatty liver disease (NAFLD) are chronic metabolic disorders with high prevalence and significant health impacts. Both conditions share common pathophysiological pathways including abnormal lipid metabolism and inflammation. Berberine (BBR), an isoquinoline alkaloid, is known for its beneficial effects on various metabolic and cardiovascular disorders. This study investigates BBR’s impact on AS and NAFLD through bioinformatics analysis and experimental models. This study utilized various bioinformatics methods, including transcriptome analysis, weighted gene co-expression network analysis (WGCNA), machine learning, and molecular docking, to identify key genes and pathways involved in AS and NAFLD. Subsequently an animal model of AS combined with NAFLD was established using ApoE^-/-^ mice fed a high-fat diet. The efficacy and mechanism of action of BBR were verified using methods such as hematoxylin and eosin (HE) staining, Oil Red O staining, and real-time quantitative PCR (RTqPCR). Through transcriptome analysis, WGCNA, and machine learning, this study identified 48 key genes involved in both AS and NAFLD. Function analysis revealed that the implicated genes were significantly involved in pathways like cytokine-cytokine receptor interaction, chemokine signaling, and IL-17 signaling pathway, suggesting their role in inflammation and immune responses. Single cell validation identified six key genes: dual specificity phosphatase 6 (DUSP6), chemokine ligand 3 (CCL3), complement component 5a receptor 1 (C5AR1), formyl peptide receptor 1 (FPR1), myeloid nuclear differentiation antigen (MNDA), and proviral integration site of murine 2(PIM2). Finally, molecular docking and animal experiments showed that BBR significantly reduced lipid deposits and inflammatory markers in liver and aortic tissues. In conclusion, BBR can improve AS combined with NAFLD by regulating genes like MNDA, PIM2, DUSP6, CCL3, C5AR1, and FPR1, with the mechanism related to inflammation control. The findings suggest potential clinical benefits of BBR in reducing the progression of both AS and NAFLD, warranting further investigation.

## Introduction

Atherosclerosis (AS) and non-alcoholic fatty liver disease (NAFLD) are both chronic metabolic disorders that pose significant clinical challenges due to their high incidence, the difficulty in prevention, and prolonged impact. AS is the leading cause of death from cardiovascular diseases [1], while NAFLD is the most common chronic liver disease [2]. Numerous meta-analyses and epidemiological evidence have demonstrated a strong correlation between their pathogenesis and progression [3–5]. NAFLD can increase the risk of cardiovascular diseases, which are the primary cause of death in patients with NAFLD.

Common risk factors NAFLD and AS include obesity, T2DM, hypertension, and dyslipidemia, most of which are components of metabolic syndrome [6, 7]. NAFLD is considered a hepatic manifestation of metabolic syndrome, while AS is a manifestation within the cardiovascular system. Therefore, lipid-lowering therapy is a common strategy for both diseases [2, 6]. Research shows that statin treatment has a protective effect on steatosis, steatohepatitis, and fibrosis, thereby improving NAFLD, and above all, substantially reducing associated cardiovascular disease morbidity and mortality [8]. However, recent Mendelian randomization studies concerning lipid-lowering drug target genes and their relationship with NAFLD have shown that targets such as HMG-CoA reductase, PCSK9, Apolipoprotein B100, LDL Receptor, and ANGPTL3 do not positively influence the outcomes of NAFLD or the risk of cardiovascular diseases [9]. This indicates that the pathophysiology behind the association of NAFLD with AS is not fully understood and may involve other pathways beyond insulin resistance, such as low-grade inflammation, oxidative stress, and the effects of disturbances in gut microbiota.

Berberine (BBR), an isoquinoline alkaloid derived from the Chinese herb Coptis chinensis and other Berberis plants, exhibits a broad spectrum of pharmacological effects. It is used to treat various diseases, including cancer, and digestive, metabolic, cardiovascular, and neurological disorders. Extensive research has shown that BBR can improve NAFLD by regulating the gut microbiome, ameliorating insulin resistance, modulating lipid metabolism, reducing oxidative stress, and suppressing inflammation [10]. Moreover, BBR can improve AS through enhancing endothelial function, antioxidative, anti-proliferative, anti-inflammatory effects, and regulating lipid metabolism [11]. Although BBR shows similar mechanisms of action in both AS and NAFLD, the specific roles and mechanisms in their co-morbidity require further investigation and clarification.

This study utilized various bioinformatics tools to analyze transcriptome and single-cell datasets from the Gene Expression Omnibus (GEO) database related to AS and NAFLD, aiming to identify pivotal genes and underlying mechanisms of AS combined with NAFLD. Key genes identified underwent molecular docking to explore BBR’s potential impacts on AS combined with NAFLD. Finally, the vitro experiments demonstrated that BBR could significantly reduce aortic plaque area, alleviate hepatic lipid deposition, and downregulate the transcription levels of MNDA, PIM2, DUSP6, CCL3, C5AR1, and FPR1, thereby improving the pathological state of AS combined with NAFLD.

## Materials and methods

### Microarray data collection and differential gene expression analysis

Original expression profile datasets of AS patients and NAFLD patients were downloaded from the GEO database (https://www.ncbi.nlm.nih.gov/geo/), including the GSE100927 dataset (platform: GPL17077, consisting of 35 healthy arterial tissue samples and 69 AS arterial tissue samples) and the GSE89632 dataset (platform: GPL14951, including 24 healthy liver tissue samples and 39 NAFLD samples). The "limma" package in R software was used to analyze differential gene expression in both datasets, with the cutoff criteria being a P-value < 0.05 and |logFC| > 0.5.

### Weighted gene co-expression network analysis and identification of key module genes

The "WGCNA" package [12] was used to construct an unweighted co-expression gene network for the GSE100927 and GSE89632 datasets. Before the WGCNA analysis, the raw read counts were first normalized using the limma and tidyverse packages. To further stabilize the variance across the mean, the normalized counts were transformed to log2-counts per million using the limma package. The median absolute deviation (MAD) of each gene in both datasets was calculated, and genes within the top 5000 MAD were selected. The "goodSamplesGenes" function was used to check for missing entries, entries with weights below the threshold, and zero variance genes in the data, returning a list of samples and genes that met the criteria for the maximum number of missing values or low weight values. Additionally, the correlation between the module eigengenes and the sample traits matrix was calculated, and the "labeledHeatmap" function was used to visualize the aforementioned correlation matrix and p-values. The modules with the most significant positive and negative correlations with the traits were selected. Finally, the key module genes and common differential genes from both datasets were merged to obtain the Common genes (CGs).

### Machine learning

Least absolute shrinkage and selection operator (LASSO) regression analysis was conducted on the CGs using the "glmnet" package. This method was employed to refine the selection of candidate biomarkers in patients with both AS and NAFLD, ultimately identifying the LASSO genes.

### Functional enrichment analysis

Using the jvenn website tool (https://jvenn.toulouse.inrae.fr/app/example.html), an intersection of the previously identified differential genes was performed to obtain the CGs. These CGs were then subjected to Gene Ontology (GO) and Kyoto Encyclopedia of Genes and Genomes (KEGG) pathway enrichment analysis using packages such as "org.Hs.eg.db," "GOplot," "enrichplot," and "clusterProfiler." A *p*-value threshold of < 0.05 was considered significant for enrichment. Additionally, the results of the enrichment analysis were visualized using packages "ggplot2", and "circlize" displaying the findings through circular and bubble charts.

### Single cell RNA sequencing (scRNA-seq) data processing

Single-cell RNA sequencing data from aortic and liver tissues were analyzed using the R statistical software and the Seurat v4.0 toolkit. Liver scRNA-seq data were obtained from the GEO dataset GSE115469 (platform: GPL16791, primary liver samples from 5 patients), and aortic scRNA-seq data from the GEO dataset GSE260657 (platform: GPL21290, including samples from 7 asymptomatic patients and 8 AS patients). After filtering out low-quality cells based on defined thresholds (200 < nFeature RNA < 10,000, mitochondrial gene percentage < 10%, nCount RNA < 600,000), data underwent initial normalization. Batch effects were mitigated using the LogNormalize method (features = 1500) and the Harmony function (max.iter Harmony = 30). Dimension reduction was conducted using t-SNE. Cell clusters were automatically annotated using the "SingleR" package. Subsequent analysis focused on the expression of LASSO genes in different cell populations within liver and aortic tissues, further narrowing down to Key Genes.

### Molecular docking

Download the 3D structures of Key genes from the PDB database and the 2D structure of BBR from Pubchem, saving it as a component ligand in SDF format. Process the protein using AutoDockTools 1.5.6 software. After preprocessing the receptor and ligand, set up the docking box to encompass all protein structures. Configure the docking parameters for the active site using the Vina algorithm. Obtain the docking results from Vina docking, which reveal the binding energy. Use PyMoL to generate images of the molecular docking interactions.

### Animal experiment

Eighteen specific pathogen-free grade male APOE^-/-^ mice, approximately 6 weeks old and weighing 18–25 g, were purchased from Beijing Vital River Laboratory Animal Technology Co., Ltd., with the production license number: [SCXK (Beijing) 2021–0006]; they were bred in the experimental animal center of the Sanyuanli Campus of Guangzhou University of Chinese Medicine, with the animal use license number SCXK (Guangdong) 2018–0001. The laboratory environment was controlled with temperatures between 18°C to 25°C and humidity levels of 40% to 70%. A 12-hour light-dark cycle was observed. During the whole experiment, we strictly adhered to the principles of reduction, refinement, and replacement. The 18 male ApoE^-/-^ mice were randomly divided into three groups: Control, Model, and BBR. The Control group received regular feed, while the other two groups were fed a high-fat diet for 12 weeks to establish a model of AS combined with NAFLD. After the model was established, mice in the Control and Model groups were administered 0.3 mL of saline via gavage. Mice in the BBR group were administered with BBR (100mg·kg-1·d) once daily via gavage, continuously for 12 weeks. At the end of the experiment, pentobarbital sodium was administered at a dose of 50 mg/kg for anesthesia, followed by cervical dislocation to euthanize the mice.

### Histological analysis

Aorta and liver tissue was preserved by fixation using 4% paraformaldehyde (Biosharp, China), embedded in paraffin, and sectioned. Tissue morphology was examined using HE staining. In addition, liver tissue was embedded in OCT and sectioned followed through Oil Red O staining. And all tissue sectioning and pathological staining were performed by Wuhan Servicebio Technology Co., Ltd. All sections were examined using a Pannoramic MIDI II digital pathology scanner (Jinan Danjier Electronics Co., Ltd) to assess aortic plaque, hepatocellular ballooning, and lipid deposition in liver tissue.

### Real-time quantitative PCR

Total RNA was extracted from liver tissue using TRIzol (Thermo Fisher Scientific). RNA concentration was measured using an ultramicro spectrophotometer (Implen, Germany), and RNA was reverse transcribed to DNA using the Evo M-MLV reverse Transcription premix kit (Accurate Biology). RT-qPCR was then performed using the SYBR Green Pro Taq HS Premixed qPCR kit (Accurate Biology). Finally, the relative quantification method was used to calculate the results using β-actin as the reference gene. Primer sequences are listed in the Table 1. The primers were designed through NCBI and synthesized by Sangon Biotech (Shanghai) Co., Ltd.

**Table 1:** RT-qPCR primers’ list.

Gene	Forword	Reverse
*FPR1*	AGCTGTTGGAAAGTTCAGGAGT	CCAGAACGATGTAGCCAGCA
*DUSP6*	ACGAGAATAGCAGCGACTGG	TTACTGAAGCCACCTTCCAGG
*CCL3*	CCCAGCCAGGTGTCATTTTCCT	CAGGCATTCAGTTCCAGGTCA
*C5AR1*	TGACCAATGAGCACCTCCAG	GCTGTTATCTATGGGGTCCATGT
*PIM2*	ATGTCTCCCCAGATTGCTGT	GGGTCCCCTCACCAAACAAA
*MNDA*	TGAGAGCAAAGGCATCCTGG	TGGTGACCTTGATCTTGACGA
*β-actin*	TCGAGCAAGAGATGGC	TGAAGGTAGTTTCGTGGATG

### Data analysis

The data, which exhibited a normal distribution, were eloquently presented as the mean ± standard deviation (SD). A comprehensive statistical analysis was conducted using the esteemed GraphPad Prism 9.0 software. The one-way analysis of variance (ANOVA) was skillfully employed to analyze the experiment data, with a significance level of *P* < 0.05 being deemed as statistically significant.

## Results

### Transcript sequencing and differential gene analysis

**Fig 1:**
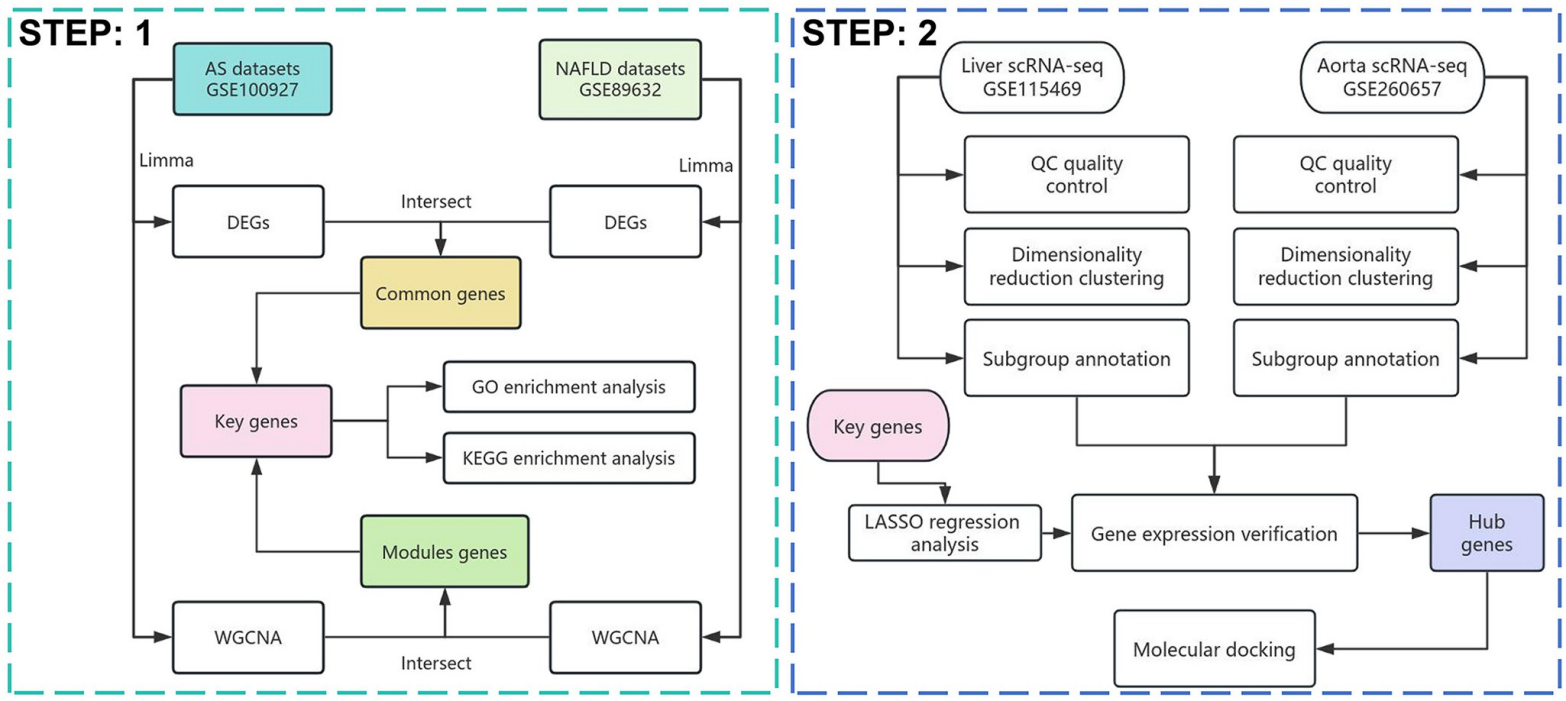
Flowchart of the study design.

**Fig 2:**
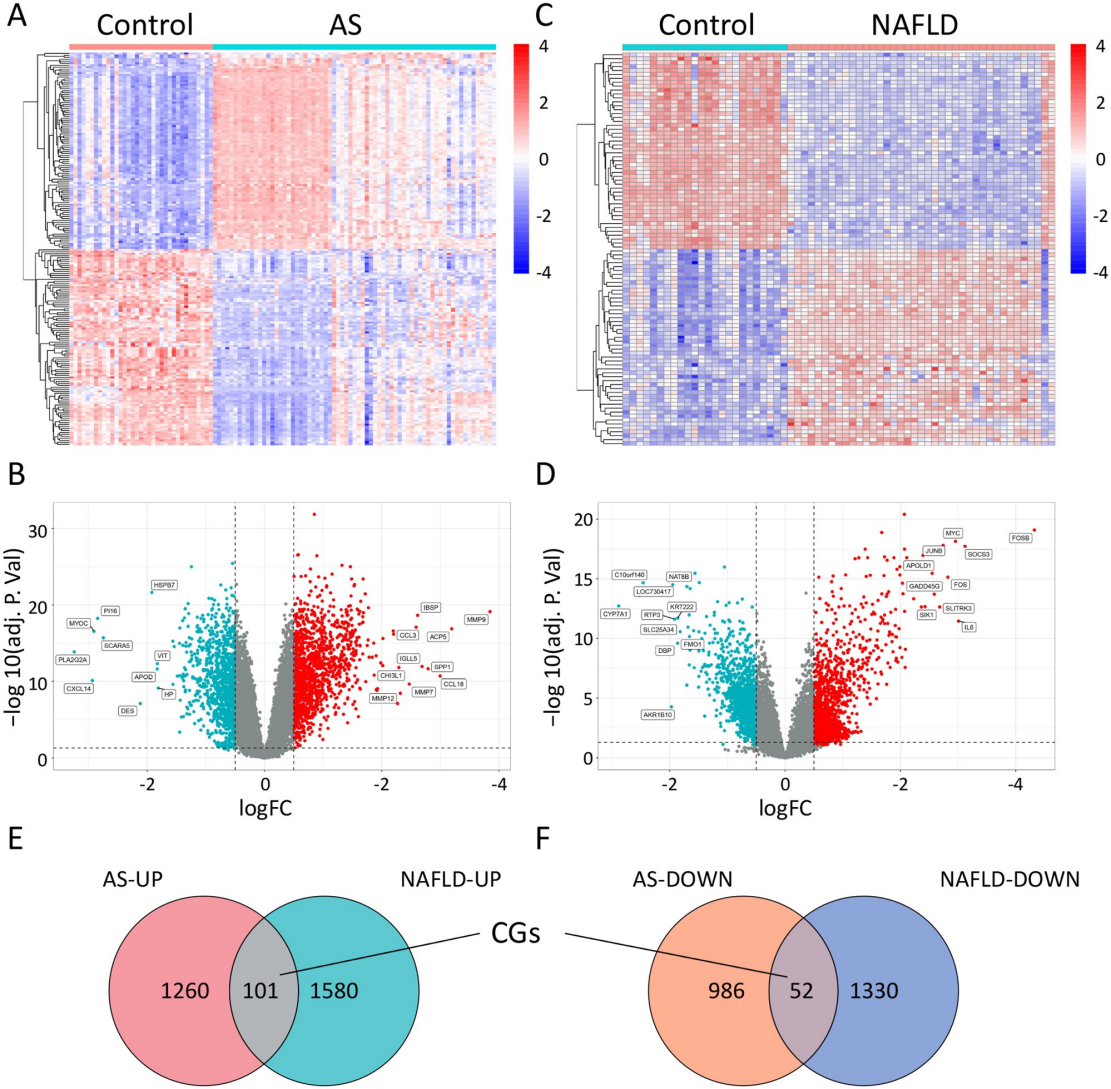
Bioinformatics of disease targets for AS and NAFLD. A-B: Heatmaps and volcano plots analyzing the differential gene expression between AS patients and normal control groups. C-D: Heatmaps and volcano plots for the differential gene expression analysis between NAFLD patients and normal control groups. E-F: Venn diagrams showing the overlap of commonly upregulated and downregulated genes between AS and NAFLD conditions.

The strategy of bioinformatics analysis is performed as shown in Fig 1. In the GSE100927 dataset, the AS group compared to the Control group revealed 2,399 differential genes, with 1,038 downregulated DEGs and 1,361 upregulated DEGs (Fig 2A and 2B). In the GSE89632 dataset, the NAFLD group compared to the Control group showed 3,063 differential genes, with 1,382 downregulated and 1,681 upregulated DEGs (Fig 2C and 2D). The intersection of genes that are downregulated and upregulated in two diseases yielded 52 commonly downregulated genes and 102 commonly upregulated genes, totaling 153 common genes. (Fig 2E and 2F).

### Selection of key targets for AS combined with NAFLD using WGCNA and machine learning

**Fig 3:**
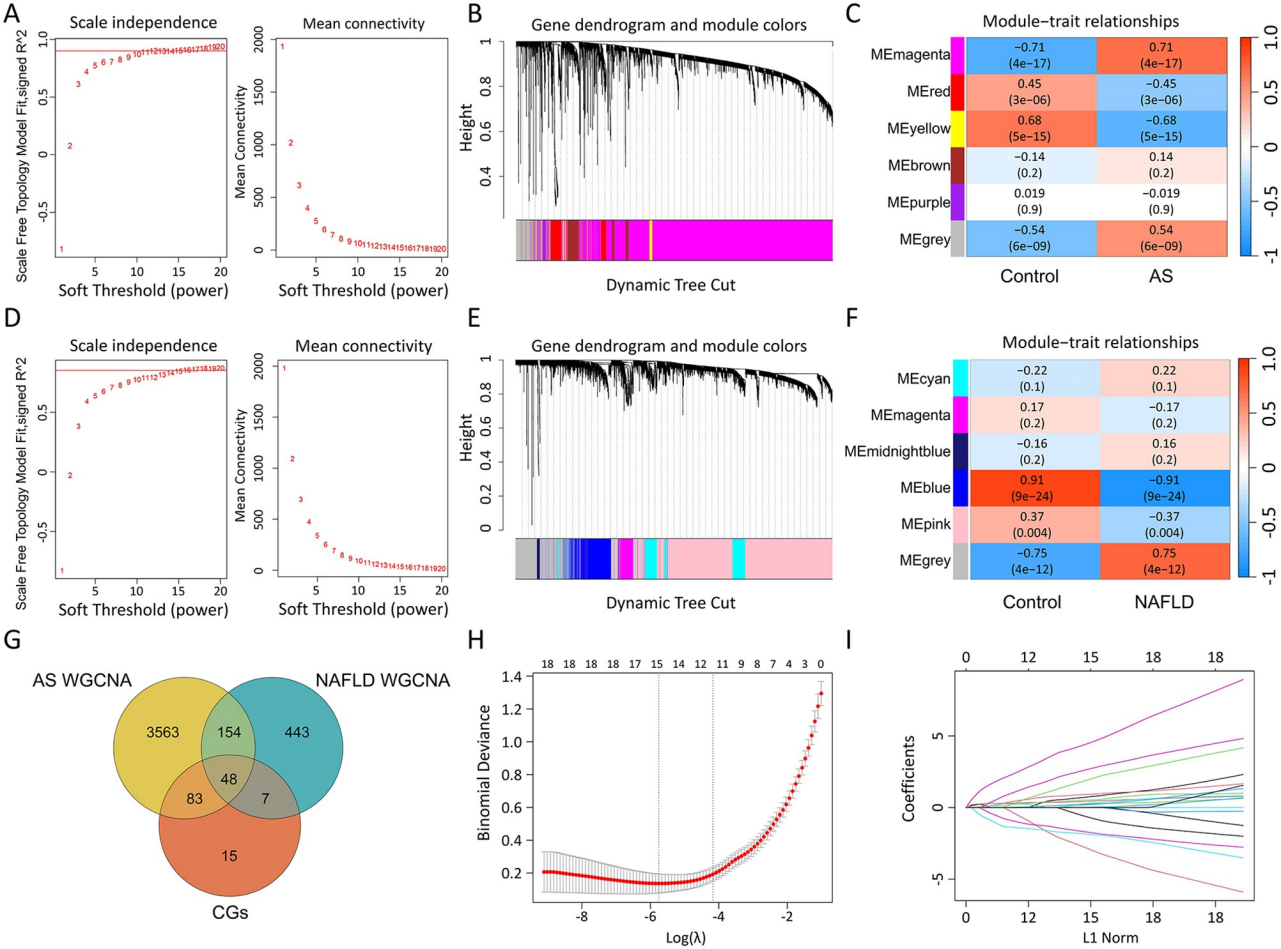
WGCNA and machine learning algorithms. A: Scale-free topology fit index and average connectivity analysis for the GSE100927 dataset. B: Dendrogram of the GSE100927 dataset, where the upper part shows the hierarchical clustering of genes, and the lower part shows gene modules, also known as network modules. C: Heatmap of gene correlations with the AS phenotype in the GSE100927 dataset. The color blocks on the left represent modules, and the color bars on the right indicate the range of correlation; the darker the color in the heatmap, the higher the correlation, with red indicating positive correlation and blue indicating negative correlation. Each cell contains numbers representing correlation strength and significance. D: Scale-free topology fit index and average connectivity analysis for the GSE89632 dataset. E: Dendrogram of the GSE89632 dataset, displaying clustering of genes into modules. F: Heatmap of gene correlations with the NAFLD phenotype in the GSE89632 dataset. G: Venn diagram of the intersection of key module genes from AS and NAFLD with CGs, yielding 48 Key Genes. H-I: Lasso regression analysis on the 48 Key Genes to compute diagnostic biomarkers’ lambda (λ) value (H) and its minimum value (I).

In the WGCNA analysis of the GSE100927 dataset, the optimal soft threshold was 10 resulting in the generation of 6 modules. Among these, the magenta module showed the highest positive correlation with AS (3848 genes, r = 0.71, *p* = 4e-17) (Fig 3A–3C). In the GSE89632 dataset, the optimal soft threshold was 16, also leading to 6 modules. Here, the blue module had the highest positive correlation with NAFLD (652 genes; r = 0.91, *p* = 9e-24) (Fig 3D–3F). An intersection of genes from these two modules with the CGs yielded 48 Key Genes (Fig 3G). Further refinement through LASSO regression analysis on these 48 Key Genes identified 15 lasso genes (Fig 3H and 3I).

### Functional enrichment analysis of CGs

To better understand the functions and specific mechanisms of these CGs, functional enrichment and KEGG pathway analyses were conducted on the 48 genes. GO terms for biological processes (BP) revealed that the CGs are primarily enriched in areas such as "leukocyte chemotaxis", "cell chemotaxis", "neutrophil chemotaxis", "neutrophil migration", "chemokine-mediated signaling pathway" and "response to lipopolysaccharide". In terms of cellular component (CC), these pathogenic genes are mainly located in "secretory granule membrane", "tertiary granule" and "tertiary granule lumen". Regarding molecular function (MF) analysis, results highlighted that "immune receptor activity", "cytokine activity", "cytokine receptor binding", "chemokine activity", "receptor ligand activity" and "Toll-like receptor binding" are the most relevant activities associated with the CGs (S1A Fig). KEGG pathway analysis showed that CGs are closely related to "Cytokine-cytokine receptor interaction", "Viral protein interaction with cytokine and cytokine receptor", "Chemokine signaling pathway" and "IL-17 signaling pathway" (S1 Fig).

### Single cell sequencing validation of lasso genes in liver and aortic tissue

**Fig 4:**
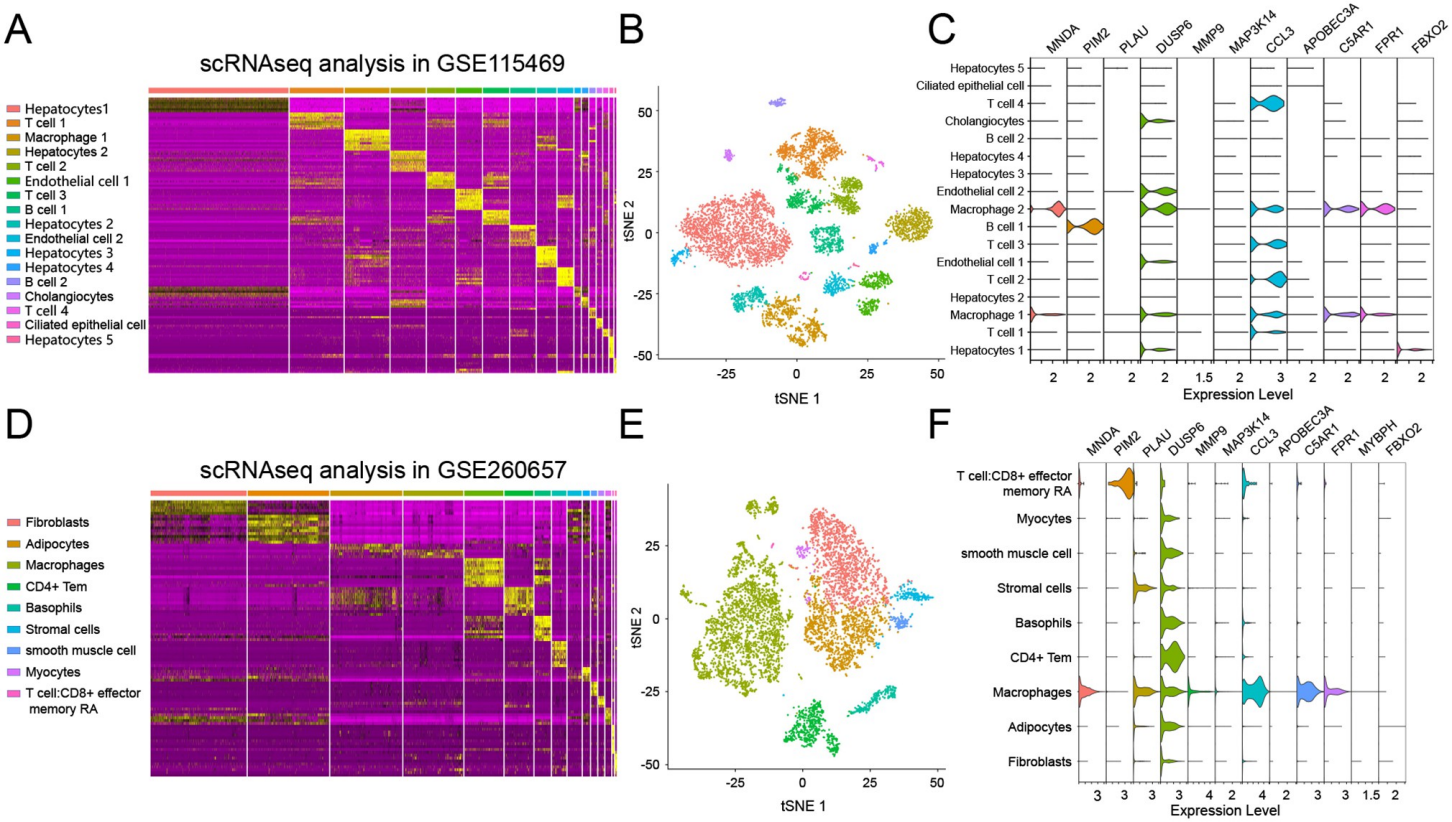
Single-cell sequencing validation lasso genes in liver and atherosclerotic aortic tissues. A: Heatmap of highly variable genes in the liver tissue single-cell dataset GSE115469, used for clustering cells. B: t-SNE cell clustering in the GSE115469 dataset. C: Violin plots showing the expression levels of Lasso genes across different liver tissue cell clusters. D: Heatmap of highly variable genes in the aortic tissue single-cell dataset GSE260657. E: t-SNE cell clustering in the GSE260657 dataset. F: Violin plots showing the expression levels of Lasso genes across different aortic tissue cell clusters.

Using single-cell RNA sequencing data from liver tissues of five patients in the GSE115469 dataset, quality control and batch correction processes were performed, yielding 8,444 cells suitable for downstream analysis. After dimensionality reduction, all cells were divided into 16 clusters based on the variability of highly variable genes (Fig 4A). Cell clusters identified via t-SNE included "Hepatocytes", " Macrophage", "Endothelial cells", "T cells", " cholangiocytes " (Fig 4B). Analysis of the expression levels of lasso genes within these clusters revealed that genes such as MNDA, PIM2, DUSP6, CCL3, C5AR1, FPR1, and FBXO2 are expressed in liver cells (Fig 4C).

Similarly, using data from aortic tissues in the GSE260657 dataset, after quality control and batch correction, we obtained 7,064 cells for downstream analysis. Post-dimensionality reduction, all cells were divided into 9 clusters based on the variability of highly variable genes (Fig 4D). Cell clusters identified via t-SNE included "Fibroblasts", "Adipocytes", "Macrophages", "CD4+ Tem", "Basophils", "Stromal cells", "Smooth muscle cells", "Myocytes", and "T cell:CD8+ effector memory RA" (Fig 4E). Analysis of the expression levels of lasso genes within these clusters revealed that genes such as MNDA, PIM2, PLAU, MMP9, DUSP6, CCL3, C5AR1, and FPR1 are expressed in aortic cells (Fig 4F).

### Distribution of core genes in liver and aortic tissue cell clusters

**Fig 5:**
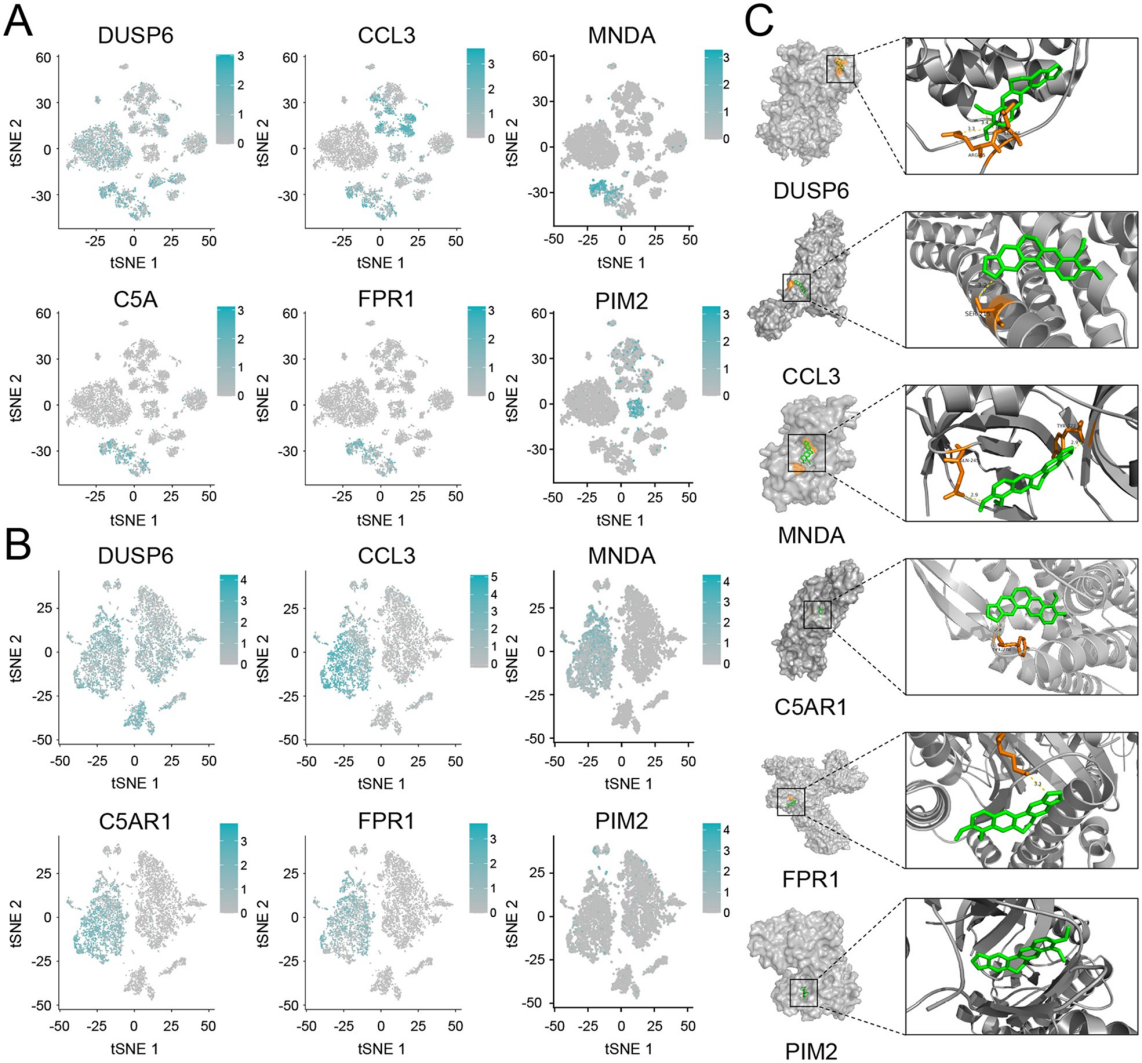
Distribution of 6 core genes in liver and aortic tissues and their molecular docking with BBR. A: The distribution of the 6 core genes in liver tissue. B: The distribution of the 6 core genes in aortic tissue. C: Results of the molecular docking of the 6 core genes with BBR.

In the liver single-cell dataset, the expression of six genes—MNDA, PIM2, DUSP6, CCL3, C5AR1, and FPR1—was analyzed. These genes were primarily expressed in Hepatocytes, with CCL3 also expressed in Endothelial cells and DUSP6 additionally found in Skeletal muscle (Fig 5A). In the aortic tissue single-cell dataset, while PIM2 was highly expressed in T cell:CD8+ effector memory RA, the other five genes—MNDA, DUSP6, CCL3, C5AR1, and FPR1—were mainly expressed in Macrophages (Fig 5B). This distribution underscores the distinct roles these genes may play in different cell types within the respective tissues.

### Molecular dock of key genes with BBR

The molecular docking results between the genes MNDA, PIM2, DUSP6, CCL3, C5AR1, FPR1, and the molecule BBR indicate effective binding interactions, as all obtained binding energies are less than -5 kJ/mol, suggesting spontaneous and stable molecular binding. Specifically: MNDA and BBR showed a docking binding energy of -6.9 kJ∙mol^-1^, PIM2–10.6 kJ∙mol^-1^, DUSP6–5.0 kJ∙mol^-1^, CCL3–7.1 kJ∙mol^-1^, C5AR1–8.5 kJ∙mol^-1^, FPR1–6.3 kJ∙mol^-1^ (Fig 5C). These results suggest that the molecular interaction between these genes’ proteinucs and BBR is not only feasible but also potentially stable and significant, offering a foundation for further exploration in therapeutic contexts.

### In vivo experiment of BBR treatment for AS combined with NAFLD

**Fig 6:**
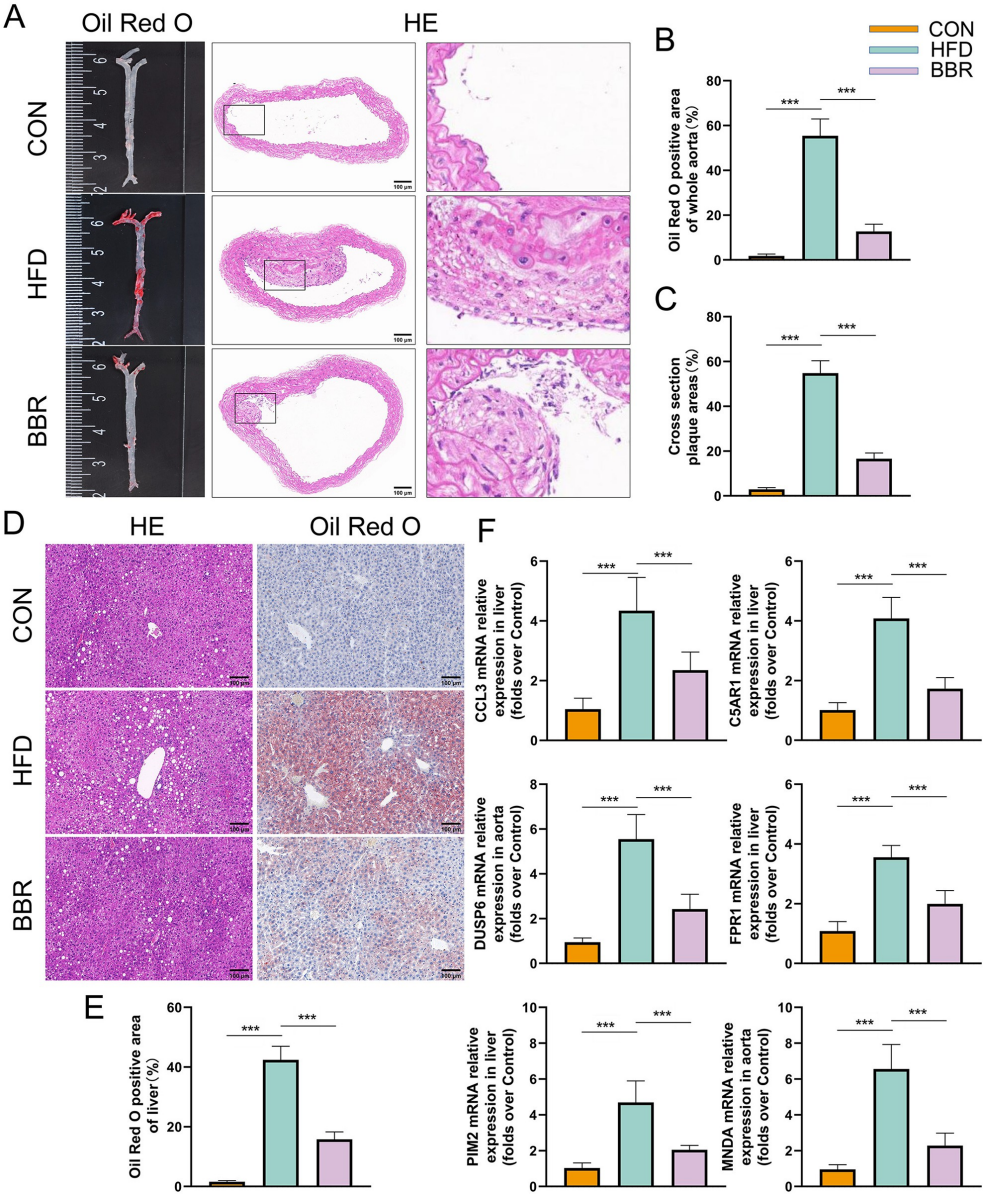
Vivo experiment validating BBR’s therapeutic effects on AS combined with NAFLD. A: Representative images of whole aorta Oil Red O staining and aortic cross-section HE staining for each group of mice (scale bar: 100 μm). B-C: Statistical graphs of whole aorta Oil Red O and HE staining for each group (n = 3). D: Representative images of liver HE staining and Oil Red O staining for various groups of mice (scale bar: 100 μm). E: Statistical graph of liver Oil Red O staining results for each group (n = 3). F: mRNA expression levels of the 6 core genes in liver tissue (n = 5).

In vivo experiments involving ApoE^-/-^ mice revealed significant pathological changes in the aorta and liver due to high-fat diet (HFD) feeding, and the therapeutic effects of BBR. Whole aorta Oil Red O and HE staining showed extensive lipid deposition and plaque formation in HFD-fed mice compared to controls. Treatment with BBR significantly alleviated the extent of aortic deposition and inhibited plaque formation in ApoE^-/-^ mice (Fig 6A–6C). HE and Oil Red O staining of liver tissues displayed noticeable steatosis and lipid accumulation, characteristic of balloon-like changes. BBR treatment markedly suppressed these alterations (Fig 6D–6E). RT-qPCR analysis of liver tissue RNA indicated that the transcription levels of MNDA, PIM2, DUSP6, CCL3, C5AR1, and FPR1 were significantly elevated in mice with AS combined with NAFLD compared to the control group. BBR treatment effectively reduced the transcription levels of these genes (Fig 6F). These findings underscore BBR’s potential as a therapeutic agent in mitigating atherosclerosis and non-alcoholic fatty liver disease pathologies by influencing key gene expressions associated with these conditions.

## Discussion

Targeting shared lipid metabolism factors, clinicians often choose lipid-lowering medications to reduce blood lipid levels, stabilize plaques, inhibit liver fibrosis, and improve NAFLD and AS [13, 14]. Despite advances in lipid-lowering medications, which effectively control blood lipid levels in many patients, these treatments do not completely prevent the progression of AS and NAFLD. In atherosclerosis, although statins are highly effective in reducing low-density lipoprotein cholesterol (LDL-C) levels and decreasing cardiovascular events, non-LDL factors such as triglycerides, low high-density lipoprotein cholesterol, inflammation, and increased platelet activity also contribute to residual risk [15], which are not the primary targets of statin therapy. In NAFLD, statins are primarily used in patients with high cardiovascular risk, but concerns remain about liver damage, muscle pain, and diabetes risk [16]. This highlights that the use of lipid-lowering medications still requires support from basic experimental and large-scale clinical study data, and there is an urgent need to explore new pathomechanisms and therapeutic drugs. Based on this, we have initiated the following research.

This study provides new insights into the shared pathogenetic mechanisms of AS and NAFLD through an analysis of transcriptome sequencing datasets (GSE100927 for AS and GSE89632 for NAFLD) from the GEO public database. The differential gene analysis and key module gene screening identified 48 key genes that are pivotal in both conditions, underscoring a significant overlap in immune and inflammatory pathways. We further utilized LASSO regression analysis to refine this list to 15 highly relevant disease genes, providing a focused framework for further investigation. Significantly, scRNA-seq played a crucial role in elucidating the cellular heterogeneity. Our study utilized scRNA-seq to validate the 15 Lasso genes, discovering that DUSP6, CCL3, C5AR1, FPR1, MNDA, and PIM2 are expressed both in the liver and the aorta.

DUSP6, CCL3, C5AR1, FPR1, MNDA, and PIM2 play similar roles in inflammatory responses. DUSP6, a serine/threonine phosphatase, can dephosphorylate and inactivate MAPKs such as ERK, thereby regulating cell proliferation, differentiation, and inflammation responses [17]. Ablation of DUSP6 efficiently prevents high-fat diet-induced NAFLD through the downregulation of CYP4A and activation of the MAPK pathway [18]. Additionally, inhibiting DUSP6 expression can improve macrophage inflammatory responses and prevent polarization towards the M1 phenotype [19]. Therefore, targeting DUSP6 may improve lipid metabolism and suppress inflammatory responses, thereby ameliorating NAFLD and AS. CCL3, a chemokine, plays a key role in recruiting monocytes and T lymphocytes to sites of inflammation in atherosclerosis. It can promote macrophage migration to plaque areas, intensifying inflammatory responses and plaque formation [20]. In NAFLD, CCL3 may also be involved in the infiltration of inflammatory cells in the liver, promoting liver inflammation and fibrosis [21], which may in turn promote the development of atherosclerosis. C5AR1, a receptor for the complement component C5a—a potent pro-inflammatory mediator—can enhance the adhesion and migration of inflammatory cells in atherosclerosis, increasing inflammatory responses and plaque instability [22]. In the context of NAFLD, the interaction between C5a and C5AR1 can exacerbate liver inflammation and damage. The absence of C5AR1 can promote macrophage polarization from the M1 to the M2 phenotype through the regulation of Toll-like receptor signaling pathways, delaying liver inflammation and fibrosis [23]. FPR1, a G-protein-coupled receptor, recognizes various peptide ligands, including bacterial formyl peptides and some host-derived peptides [24]. In inflammatory responses, FPR1 regulates the migration and activation of neutrophils, macrophages, and other immune cells [25]. In atherosclerosis, FPR1 exacerbates plaque formation and instability by promoting the recruitment and activation of inflammatory cells [26]. Additionally, another subtype, FPR2, can also increase macrophage infiltration in plaques, promoting the development of atherosclerosis, but it can also increase the collagen fiber content within plaques, thereby enhancing plaque stability. Thus, FPR2 plays a dual role in atherosclerosis [26]. In NAFLD, FPR1 can regulate the expression of hnRNP U to promote lipid oxidation and activate the Akt pathway to inhibit hepatic gluconeogenesis [27], thereby improving lipid metabolism in NAFLD. Moreover, FPR1 has anti-inflammatory and anti-apoptotic roles in acute liver injury [28], although its role in chronic liver damage such as NAFLD is not yet clear. MNDA is primarily expressed in myeloid cells, such as monocytes and macrophages, and participates in the innate immune response [29]. PIM2 is a serine/threonine protein kinase involved in various cellular processes, including cell proliferation, survival, metabolism, and inflammatory responses. However, the direct role of MNDA and PIM2 in AS combined with NAFLD has not been reported. These molecules play significant roles in regulating the migration, activation, and mediation of inflammatory responses of immune cells. The production of C5a can increase the expression of CCL3, while the immune cells recruited by CCL3 can produce more complement activation products, including C5a, thus enhancing the C5AR1 signaling. These interactions enhance each other, thereby promoting the inflammatory response. FPR1 can promote the chemotaxis and activation of immune cells, further releasing inflammatory cytokines and pro-inflammatory mediators, participating in the pathological processes of NAFLD and AS.

NAFLD encompasses a broad spectrum of disease entities, ranging from simple hepatic steatosis to non-alcoholic steatohepatitis, liver fibrosis, cirrhosis, and hepatocellular carcinoma, exhibiting a state of continuous low-grade inflammation affecting the liver and systemically [30]. The early stage NAFLD is primarily characterized by substantial lipid accumulation, subsequently leading to impaired hepatocyte metabolic capacity and the accumulation of reactive oxygen species, which activates the inflammatory responses in hepatic parenchymal cells and extracellular cells such as Kupffer cells and hepatic stellate cells. Activated Kupffer cells release inflammatory mediators like interleukin-6 and tumor necrosis factor-alpha [31]. Additionally, elevated levels of high-sensitivity C-reactive protein have been found in the early stages of NAFLD patients [32]. These inflammatory factors further impair the lipid metabolism of the liver, simultaneously enter the circulatory system through the portal system, activate vascular endothelial cells, initiate inflammatory responses, and compromise vascular function and structure. This is exactly the pathogenesis of atherosclerosis. Thereby promoting the development of AS. Thus, chronic persistent inflammation may be crucial for mediating hepatic and AS.

In recent years, increasing research has focused on the benefits of BBR in cardiovascular and metabolic diseases, particularly in the treatment of AS and NAFLD. BBR’s anti-inflammatory and antioxidant effects simultaneously reduce inflammation in arterial walls and the liver, while its lipid-modulating action aids in improving overall metabolic health, thus benefiting both diseases. However, the specific role of BBR in the comorbidity of these conditions remains unknown. In this study, we used molecular docking techniques and found that BBR binds well with MNDA, PIM2, DUSP6, CCL3, C5AR1, and FPR1, suggesting that BBR may exert its effects through the regulation of these six genes. Subsequently, we constructed an AS and NAFLD animal model in ApoE^-/-^ mice fed a high-fat diet to preliminarily explore the mechanisms of action of BBR. Oil Red O and HE staining results suggested that BBR could improve lipid deposition in the aorta and liver. PCR results showed that, compared to the model group, BBR could inhibit the transcriptional expression levels of genes such as MNDA, PIM2, DUSP6, CCL3, C5AR1, and FPR1. Therefore, we revealed that BBR can improve AS combined with NAFLD by regulating genes like MNDA, PIM2, DUSP6, CCL3, C5AR1, and FPR1, with the mechanism related to inflammation control.

## Conclusion

In conclusion, we comprehensively analyzed the comorbidity mechanisms of AS combined with NAFLD through bioinformatics methods and verified the functional mechanisms of BBR on the comorbidity utilized molecular docking and in vitro experiments. These findings offer a foundation for the further development of BBR as a promising treatment strategy for patients with these co-morbid conditions. Nevertheless, further research is required to elucidate the detailed interactions among these genes and their specific contributions to disease progression and treatment outcomes.

## Supporting information

S1 Fig Functional enrichment analysis of key genes. A: The results of the Gene Ontology (GO) enrichment analysis for the 48 Key Genes are presented in a circular diagram. B: The results of the KEGG pathway enrichment analysis for the Key Genes are displayed in a bubble chart format.
(TIF)
