# Supplementary figures and images for: Exploring the mechanism of berberine treatment for atherosclerosis combined with non-alcoholic fatty liver disease based on bioinformatic and experimental study
Source: PLoS One. 2024 Dec 19;19(12):e0314961. doi: 10.1371/journal.pone.0314961 (PMC11658604; doi:10.1371/journal.pone.0314961)

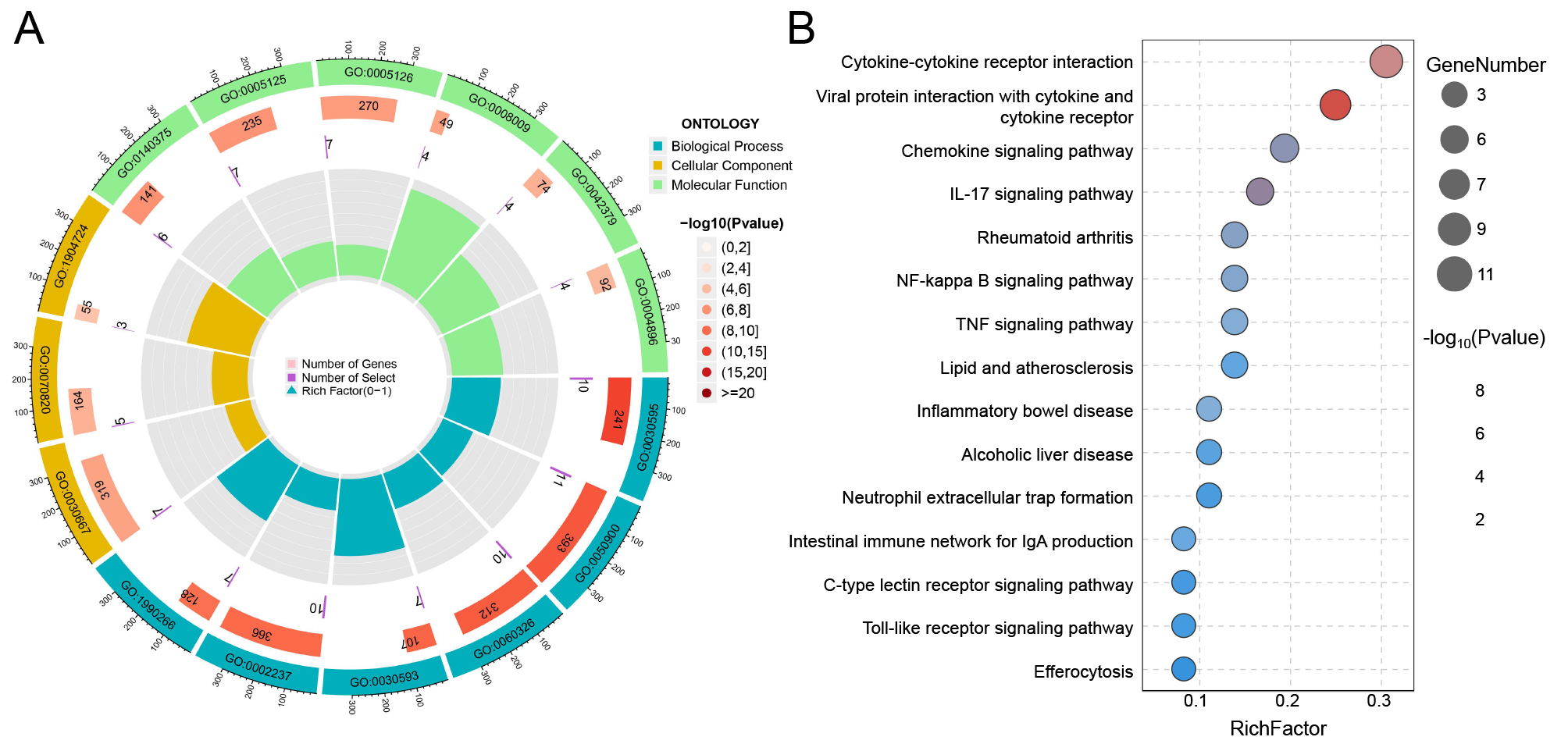

Supplement: S1 Fig — A: The results of the Gene Ontology (GO) enrichment analysis for the 48 Key Genes are presented in a circular diagram. B: The results of the KEGG pathway enrichment analysis for the Key Genes are displayed in a bubble chart format. (TIF) [file pone.0314961.s001.tif]
